# Enhancement of flavor components of oolong tea and dark tea based on graphene heating film

**DOI:** 10.1016/j.fochx.2025.102433

**Published:** 2025-04-03

**Authors:** Jiyuan Yao, Xinyuan Lin, Zihao Qiu, Xun Meng, Juan Chen, Ansheng Li, Xindong Tan, Shaoqun Liu, Peng Zheng, Binmei Sun, Hongqiang Kong

**Affiliations:** a College of Horticulture, South China Agricultural University, Guangzhou 510642, China; b Shenzhen Xuegu Industrial Co., Ltd, Shenzhen 518000, China

**Keywords:** Graphene heating film, Tea, Quality, Volatile component, GC–MS

## Abstract

Reheating is crucial for improving tea quality, and graphene heating film provides a stable, uniform heating surface. This study used graphene heating film to heat oolong and dark tea at medium (M, 65 °C) and high (H, 75 °C) temperatures for 10, 20, and 30 min to assess the impact on flavor compounds. The results showed that the optimal parameters are as follows: the content of ester catechins decreased, the content of non-ester catechins increased, and the concentrations of woody and fruity compounds (Cedrol, Limonene, trans-Isoeugenol, Indole) significantly increased at M10 or H10 in oolong tea. The ester catechin content decreased at H20, the non-ester catechin content increased at M20, and the concentration of Floral compounds (trans-β-ionone) increased at H30 in dark tea. This study explores the potential of graphene heating film in tea processing, offering a theoretical basis for new technology in tea flavor enhancement.

## Highlights

• Graphene heating film can be applied to tea refire.
• There are differences in the re-tempering parameters adapted to different teas.
• The application of graphene heating film has changed the ratio of ester to non-ester catechins in tea.
• The application of graphene heating film improves the floral and fruity aroma of tea.

## 1 Introduction

Tea quality consists of five aspects: appearance, soup color, taste, aroma and leaf bottom. Among them, flavor and aroma are the most important and decisive factors for the quality of tea. The volatile components of tea are complex and diverse, including alcohols, esters, aldehydes, ketones, alkenes, etc., which provide an important role for tea aroma, such as linalool, violet ketone provides floral aroma for tea, decanal provides fruity aroma, cedrol provides woody aroma, and all the volatile metabolites together constitute the tea aroma, and its type and content largely determine the richness and persistence of tea aroma. In terms of taste, strong bitterness and astringency is one of the reasons for lowering the quality of tea, and the bitterness and astringency are mainly caused by caffeine and catechins, which include ester catechins and non-ester catechins, the ester catechins have a heavy bitterness and astringency and contribute to a strong astringency, while the non-ester catechins have a weaker astringency and contribute to the sweetness(Liu & Tzen, 2022; Wang, Bi, et al., 2023; Zhou, He, & Zhu, 2023).

Recent studies have demonstrated that the quality of tea can be enhanced by the application of the reflame process, which has been shown to reduce the presence of stale flavors, generate a diverse array of aldehydes, ketones, and heterocyclic compounds, and enhance the woody, floral, and nutty aromas of tea(Cao et al., 2021; Fu et al., 2020; Zhan et al., 2023). Conventional reheating methods encompass two primary approaches: the use of charcoal and the utilization of microwaves(Dong et al., 2011; Wang, Liu, et al., 2023), charcoal heating has been demonstrated to reduce the bitterness of tea and enhance its aroma. However, the process of charcoal heating is not uniform, resulting in inconsistent heating and significant charcoal consumption, which can lead to elevated costs. Additionally, the combustion of charcoal releases carbon fumes, which have deleterious effects on human health and the environment(El et al., 2019; Lin & Chen, 2022); microwave penetration is robust, with heating occurring from the interior to the exterior of tea leaves. This process aims to enhance the quality of the tea leaves while concurrently preventing the occurrence of “outside scorched, inside raw” tea leaves. The heating is expeditious(Guo et al., 2004; Li et al., 2022; Yun, 2013; Zhou, Zhang, et al., 2023). However, prolonged exposure to microwaves can jeopardize human health and is potentially threatening(Hao et al., 2015; KALANT, H., 1959; Sun et al., 2017). Charcoal re-flame and microwave re-flame present significant challenges in terms of operation and safety. Conversely, graphene, a novel form of high-performance nano-material, possesses the capacity to convert electric energy into heat energy, with a heating surface that is both even and stable. The heating mechanism of graphene involves far-infrared radiation, emitting a wavelength range of 6–14 μm, which is well-aligned with the human body's infrared spectrum between 2 and 25 μm. This characteristic of far-infrared light has garnered the moniker “life light wave” within the healthcare sector, underscoring its significance in biological applications(Chu et al., 2023; Zhang et al., 2017). It has also been shown that far-infrared reheating teas have a more pronounced nutty aroma than normal blast reheating teas(Zhu et al., 2021). At present, most of the domestic and foreign use of electric heaters or far-infrared quartz tube for infrared heat source on the tea reflame aroma process, which exists in the low utilization of heat energy, temperature changes in the range of large, and other shortcomings(Liu et al., 2021; Xiang et al., 2021). Graphene heating film can be used to adjust the heating power and temperature by temperature control switch with small variation, which provides great convenience for users to adjust the reheating temperature of tea(Chaudhari et al., 2024; Liu et al., 2021; Xiang et al., 2021). Moreover, there have been products on the market that utilize graphene in the context of tea, including a graphene health kettle, a graphene roaster, and a graphene wake-up tea, among others. These products aim to enhance the flavor quality of tea services. However, research on the impact of different types of tea and different graphene heating parameters is currently limited. The absence of scientific data to support this field hinders the innovation of new technology and new equipment in the tea refining process.

In recent years, the market for oolong and dark teas has undergone significant expansion. Rougui tea, produced in Wuyi Mountain in Fujian Province, China, is renowned for its distinctive cinnamon aroma. Puerh raw tea, cultivated in China's Yunnan Province, is highly regarded by consumers for its strong aroma and reported health benefits(Lin et al., 2024; Ma et al., 2023). On the research to enhance the quality of oolong and dark teas, Wei (Wei et al., 2023) et al., heat-treated dark tea and found that heating significantly weakened the musty flavor of aged tea. Wang(Wang, Xiao, et al., 2023) et al.'s heat treatment of oolong tea showed that heating could reduce the green flavor of oolong tea and stimulate the floral and fruity aroma and caramel aroma. The roasting process is pivotal in the development of the distinctive aroma of Rougui tea, while Puerh raw tea exhibits a more pronounced grassy flavor due to its lack of roasting. Consequently, Rougui tea and Puerh raw tea were selected as the representatives of oolong tea and dark tea, respectively, for the purpose of this study. The objective of the study was to explore whether the floral and fruity aroma of the tea can be further stimulated by heating to enhance the quality of the tea(Lin et al., 2024).

In this study, oolong tea and dark tea were selected as experimental materials, and the untreated tea samples were used as the control (CK), different grades (M, H) and different times (10, 20, 30 min) were heated. The caffeine, catechins and volatile components of tea were determined by HPLC and GC–MS detection methods, and the effect of graphene heating film on improving the quality of different teas and the heating parameters with better effects were explored, which will play a certain positive significance in providing a theoretical basis for innovating the tea reheating and aroma extraction process.

## 2 Materials and methods

### 2.1 Materials

The dark tea samples utilized in this experiment were Pu'er (2022, Mansong Shengpu, Tianhong Tea Industry, Kunming, Yunnan Province, China) and oolong tea samples were Rougui tea (2022, Jinzun Rougui, Fuzhou City, Fujian Province, China). The heating process was conducted using a graphene heating film at mid-range levels (65 °C, M) and high-range levels (75 °C, H), with 0 min designated as the control. The duration of the heating process was set to 10 mins, 20 mins, and 30 mins for the two tea samples, i.e., oolong and dark tea. The treatments corresponded to the number of the control (CK), the mid-range treatment of 10 mins (M10), mid-range treatment 20mins (M20), mid-range treatment 30mins (M30), high-grade treatment 10mins (H10), high-grade treatment 20mins (H20), and high-grade treatment 30mins (H30). The treated tea leaves were ground into a powder and preserved for the purpose of determining the caffeine, catechins, and volatile compounds present in the tea. For the determination of each index, three samples were taken to repeat the experiment to ensure the reliability of the experiment.

### 2.2 Determination of catechins

The extraction and analysis of catechins was conducted using a high-performance liquid chromatography (HPLC) system, model Waters Alliance E2695, equipped with a 2489 UV/Vis detector (Waters Technologies). The procedure was executed in accordance with the Chinese national standard protocol (GB/T 8313–2018).A quantity of 0.2 g of tea powder was added to 8 mL of 70 % methanol (*v*/v), which served as the extraction solution. The resultant filtrate (1 mL) was then filtered through a 0.22-μm nylon membrane filter, and 10 μL of each filtered filtrate was injected onto an XSelect HSS C18 SB column (250 mm × 4.6 mm, 5 mm, Waters Technologies). The catechin monomers were eluted with a 0.1 % formic acid aqueous solution (v/v) (A) and 100 % acetonitrile (B) as mobile phases.The gradient was set at 8 % B for the first 5 min; gradually adjusted to 75 % B for 5 to 14 min; and gradually reached 8 % B for 14 to 30 min. The detection wavelength was set at 280 nm.

### 2.3 Determination of caffeine

According to the method of Jiahao Chen et al.(Chen et al., 2023), 0.45 g of magnesium oxide and 0.1 g of tea powder were added to 30 mL of ultrapure water at 100 °C and ultrasonically agitated in a water bath for 30 min to extract caffeine. A volume of 1 mL of the extract was filtered through a 0.22-μm nylon membrane filter and analyzed by HPLC (Waters Alliance E2695) equipped with a 2489 UV/Vis detector (Waters Technologies, Milford, MA, USA). The analysis utilized an XSelect HSS C18 SB column (4.6 × 250 mm, 5 mm) (Waters Technologies, Milford, MA, USA)., MA, USA) at 35 ± 1 °C with 100 % ultrapure water as solvent A and 100 % methanol as solvent B. The compounds were eluted for 14 min at an isocratic pressure of 70 % A and 30 % B at a flow rate of 0.9 mL/min with a detection wavelength of 280 nm.

### 2.4 Determination of volatile substances

Volatiles were identified and quantified by headspace gas chromatography, as previously described by Li et al.(Li et al., 2024). This analysis was conducted using an Agilent 7890B gas chromatograph and a 5977 A mass spectrometer (Agilent, Santa Clara, CA, USA), along with an HP-5 mass spectrometry column (30 m × 0.25 mm × 0.25 μm film thickness), and applying a headspace solid-phase microextraction (HS-SPME). The headspace solid-phase microextraction (HS-SPME) method was used to extract the volatile substances from the tea leaves. The solid-phase microextraction device utilized was composed of divinylbenzene/carboxylic acid/polydimethylsiloxane (DVB/CAR/PDMS) fibers (inner diameter 50/30 μm, length 2 cm; Supelco, Darmstadt, Germany) was inserted into the headspace vial. The compounds were extracted at 80 °C for 40 mins. After extraction, the SPME fibers were inserted into a GC–MS at 250 °C for 3 mins.

The column flow rate was set at 1.0 mL/min, the carrier gas was high-purity helium (purity≥99.99 %), and the solvent delay time was 4 min.The initial temperature was set at 50 °C for 1 min, and the temperature was increased to 220 °C a rate of 5 °C/min for 5 min. The ion source temperature was set to 230 °C, and the electron impact (EI) ionization source was operated at 70 eV.The scanning range was set from 30 to 400 amu.

### 2.5 Calculation of odor activity values (OAVs)

OAV is the ratio of the concentration of an aroma component in an aqueous solution to its odor threshold. The OAV method was used to evaluate the contribution of each volatile compound to the aroma of the tea samples. The OAV_i_ of each volatile compound was calculated as OAV_i_ = C_i_/T_i_, where C_i_ is the concentration of compound i (μg·kg^−1^) and T_i_ is the threshold of compound i (μg·kg^−1^).

### 2.6 Calculation of volatile substance characterization

Quantification of volatile compounds (μg/kg) was based on a comparison of peak areas in samples and ethyl decanoate. Compound identification was achieved by cross-referencing the compounds' retention indexes (RI), determined using n-alkanes C9-C21, with the National Institute of Standards and Technology (NIST) MS database (https://webbook.nist.gov/). The RI was calculated using the formula: RI = 100n + 100[RT(x)-RT(n)] / [RT(n + 1)-RT(n)], where RT(x) represents the retention time of compound x, and RT(n) and RT(n + 1) are the retention times of the n-alkanes preceding and following the elution of the compound. Compound matches were accepted when the calculated RI was less than 15, or if the mass spectral match factor was more than 90.

### 2.7 Data analysis

Microsoft Office Excel 2021 was utilized to organize, summarize, and calculate the sample datas, while SPSS 27 (SPSS Inc., Chicago, IL, USA) was used to apply two-way ANOVA and Turkey HSD statistical methods to analyze the effects to non-volatile substances and volatile substances of tea by different times and different treat levels. The affected non-volatile and volatile substances in tea were analyzed for differences, and *p* < 0.05 was considered significant. The statistical analysis and visualization of non-volatile and volatile compounds were performed using GraphPad Prism 9.5.0 software (GraphPad Software, Inc., La Jolla, CA). Partial Least Squares Discriminant Analysis (PLS-DA) and Projected Variable Importance (VIP) analyses were performed using SIMCA (Version 14.1, Umetrics, Umea, Sweden) software, and heat maps were produced using Tbtools II software(v2.152, Guangzhou, China).

## 3 Results

### 3.1 Changes in non-volatile substances

**Fig. 1:**
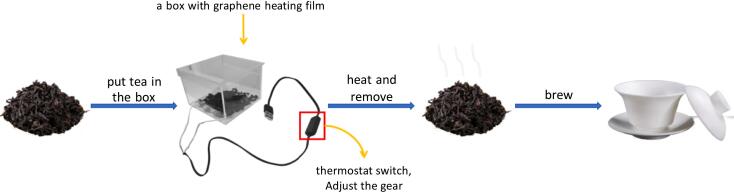
Graphene Heating Film Usage.

**Fig. 2:**
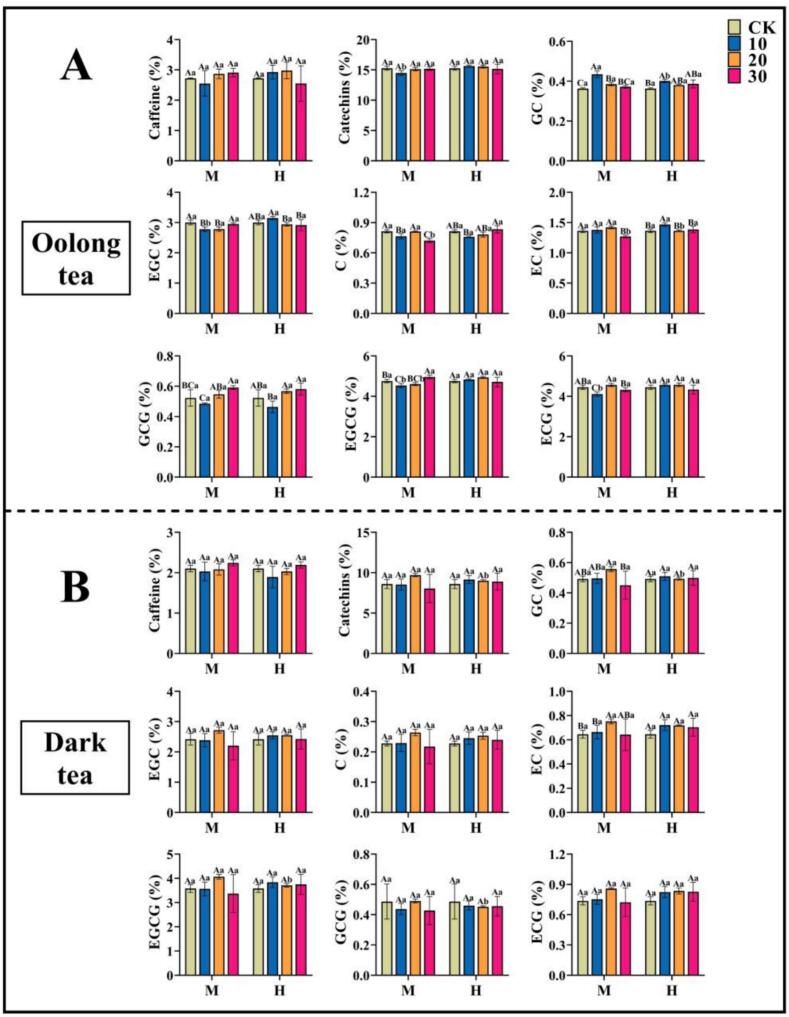
Differences in dry weight of non-volatile matter in different combinations. (A) Non-volatile matter content of oolong tea. (B) Non-volatile matter content of dark tea. The letters on the bar graphs represent significant levels, where capital letters indicate: differences in the effects of different heating times on tea under the same gear heating; and lower case letters indicate: differences in the effects of different heating gears on tea under the same time heating. Error bars represent the mean ± SD and letters (A, B, C, a, b, c) indicate the differences obtained according to the Turkey HSD comparison method (*p* < 0.05), *n* = 3.

Compared with CK, the heating treatment had no significant effect on the caffeine content of oolong tea and dark tea, both in terms of gear and time. There was no significant difference in the catechins of oolong tea and dark tea at the same gear; while the catechin content of oolong tea M10 was significantly lower than that of H10, and that of dark tea H20 was significantly lower than that of M20 at the same time. Regarding the ester catechins, the ester catechins (GCG, EGCG, and ECG) of oolong tea were significantly lower at the same gear of M at the heating time of 10 mins, and the GCG content was 7.14 % lower, and the EGCG content was 4.6 % lower, compared with that of CK. Compared with CK, the content of GCG decreased by 7.14 %, EGCG by 4.6 % and ECG by 7.7 %. At the same time, the content of EGCG of oolong tea was significantly lower than that of H10 and H20 at M10 and M20, and the content of ECG was significantly lower than that of H10 at M10. At the same time, the content of ester catechins (GCG, EGCG) of dark tea was significantly changed at the heating time of 20 mins, and the content of GCG and EGCG at H20 decreased by 7.9 % and 8.7 %, respectively, compared with that at M20. Regarding the non-ester catechins, under the same gear, the GC contents of oolong tea all reached the highest at 10 mins of heating, and M10 was significantly higher than H10, with an increase of 19.9 % compared with CK; the EGC and EC contents were at the highest at H10, with an increase of 4.9 % and 7.6 %, respectively, compared with CK; and the C content reached the highest at H30, with an increase of 2.5 % compared with CK, but there was no significant difference. Under H treatment, there was no significant difference between the GC and EC contents of dark tea, while under M treatment, the GC and EC contents of dark tea were at their highest at M20, and GC was significantly higher at M20 than at H20, and GC and EC were increased by 13.3 % and 16.5 %, respectively, compared with CK. There was no significant difference in EGC and C of dark tea under the same gear or time (Fig. 2A, B). In summary, the ester catechin content of oolong tea decreased at M10 or H10, while the non-ester catechin content increased. Dark tea exhibited lower ester catechin content at H20 and higher non-ester catechin content at M20. The utilization of graphene heating film has the potential to further balance the bitterness and astringency, as well as the sweetness, of tea, thereby enhancing its overall taste. (See Fig. 1.)

### 3.2 Changes in volatile substances

#### 3.2.1 Effect of graphene heating film on the aroma of oolong tea and dark tea

A gas chromatography–mass spectrometry (GC–MS) analysis was employed to detect volatile substances in oolong tea and dark tea that had been processed with a graphene heating film. A total of 68 volatile compounds were identified in oolong tea and 31 volatile compounds were identified in dark tea. The volatiles identified in oolong tea included 11 alcohols, 19 esters, 10 aldehydes, 7 alkenes, 6 ketones, and 15 other volatiles, while those identified in dark tea included 8 alcohols, 6 esters, 3 aldehydes, 2 alkenes, 5 ketones, and 7 other volatiles.

**Fig. 3:**
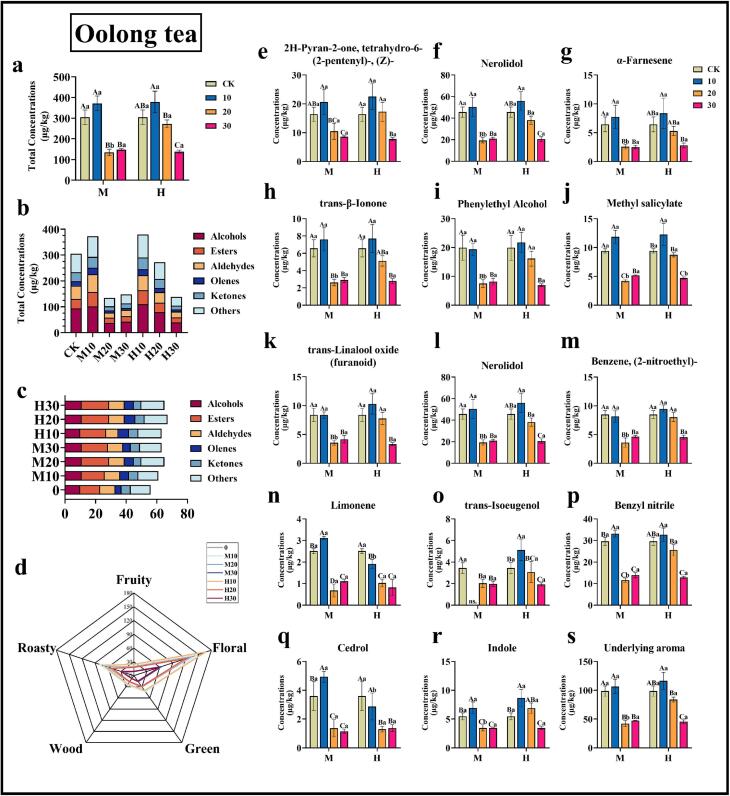
Effects of different gears and different treatment times of graphene heating film on the changes of volatile substances in oolong tea. (a) represents the total concentration of volatile substances in oolong tea under different treatments of graphene heating film; (b) represents the changes in the concentration of different types of volatile substances identified; (c) represent the changes in the quantity of different types of volatile substances identified; (d) Radar chart of aroma and flavor of oolong tea under different treatments; (e-m) represent the changes in the concentration of the nine types of volatile substances with the highest concentration identified; (l-r) Concentration of potential key aroma substances of oolong tea under different treatments; (s) Total concentrations of potential key aroma compounds in oolong tea under different treatments. The letters on the bar graphs represent significant levels, where capital letters indicate: differences in the effects of different heating times on tea under the same gear heating; and lower case letters indicate: differences in the effects of different heating gears on tea under the same time heating. Error bars indicate Mean ± SD, and letters (A, B, C, a, b, c) indicate differences obtained according to the Turkey-HSD comparison method (*p* < 0.05), *n* = 3.

**Fig. 4:**
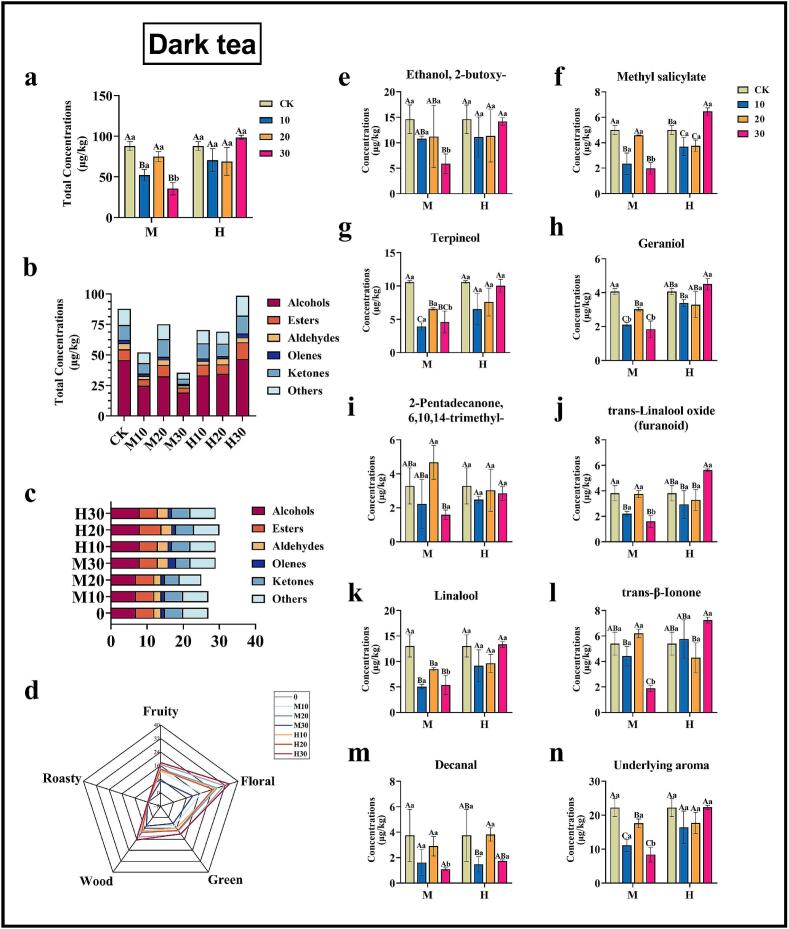
Effects of different gears and different treatment times of graphene heating film on the changes of volatile substances in dark tea. (a) represents the total concentration of volatile substances in dark tea under different treatments of graphene heating film; (b) represents the changes in the concentration of different types of volatile substances identified; (c) represent the changes in the quantity of different types of volatile substances identified; (d) Radar chart of aroma and flavor of dark tea under different treatments; (e-m) represent the changes in the concentration of the nine types of volatile substances with the highest concentration identified; (l-r) Concentration of potential key aroma substances of dark tea under different treatments; (s) Total concentrations of potential key aroma compounds in dark tea under different treatments. The letters on the bar graphs represent significant levels, where capital letters indicate: differences in the effects of different heating times on tea under the same gear heating; and lower case letters indicate: differences in the effects of different heating gears on tea under the same time heating. Error bars indicate Mean ± SD, and letters (A, B, C, a, b, c) indicate differences obtained according to the Turkey-HSD comparison method (p < 0.05), n = 3.

In the study's identification of volatile matter concentration, the total volatile matter concentration of oolong tea produced significant differences under different treatments of graphene heating film. Compared with the control group (CK), the total volatile matter concentrations of M10 and H10 were increased by 22.07 % and 24.33 %, respectively (Fig. 3a). The total volatile matter concentrations were significantly decreased in all other treatments. In dark tea, the total volatile matter concentration of H10 exhibited a modest enhancement, though this enhancement was not statistically significant (12.10 %).The remaining treatments did not result in a significant enhancement of the total concentration of volatile substances in dark tea, with M30 demonstrating the most substantial decrease (59.55 %) (Fig. 4a).

The graphene heating film had a significant effect on the number of species of volatiles and their concentrations of different types of oolong and dark teas. The concentrations of alcohols in oolong tea increased by 7.94 % and 17.15 %, respectively, after M10 and H10 treatments. In addition, the volatile substances, including esters, aldehydes, ketones, alkenes, and others, exhibited a comparable enhancement pattern in oolong tea as observed in the control group following M10 and H10 treatments. Among these substances, esters demonstrated the most pronounced enhancement, with a 53.40 % increase in concentration (Fig. 3b). In dark tea, the concentration of various types of volatile substances after different treatments exhibited a similar pattern of change with the total concentration. The esters and ketones of M20 increased by 4.03 % and 20.09 %, respectively, while the concentrations of alcohols, esters, and ketones of H30 increased by 1.76 %, 4.81 %, and 22.07 %, respectively. Conversely, the concentration of all types of substances in dark tea subjected to alternative treatments exhibited a decline (Fig. 4b).

Qualitatively, more volatile substances were identified in dark teas M30, H10, H20 and H30 compared with CK, and alcohols and esters increased by one in all of the above treatments compared with CK (Fig. 4c). However, in oolong tea, all treatment groups produced an enhancement in the number of volatile substance species relative to CK, with H20 showing the highest increase in the number of volatile substance species at 19.64 %. The results indicated that the use of graphene heating film using both high-grade and mid-range treatments for 10 mins, 20 mins, and 30 mins were able to increase the volatile compounds that could be identified and enhance the aroma levels of rich oolong tea (Fig. 3c).

The concentrations of all volatile metabolites identified in oolong and dark teas under different treatments were further normalized and then made into heat maps (Figs. S1-S2) to visualize the changes in the aroma of oolong and dark teas more intuitively during the treatments. The yellow-green color in the heat map represents the downward adjustment of the concentration of volatile substances after treatment, and the rosy red color represents the upward adjustment of the concentration of volatile substances after treatment, and the depth of the color represents the degree of upward and downward adjustment of the concentration of the substance. Similar to the results described in the previous section, for oolong tea, most of the aroma substance concentrations were up-regulated at M10 and H10, while almost all of the aroma substance concentrations were significantly decreased at M20, M30, and H30; moreover, a smaller portion of the aroma substance concentrations were up-regulated at the H20 treatment. For dark tea, the vast majority of aroma substance concentrations were up-regulated at H30; a very small portion of aroma substances were up-regulated at M20 or H10; and the rest of the treatments instead decreased the aroma substance concentrations of dark tea. The change rule of aroma substance concentration was basically consistent with the previous trend.

The nine volatiles with the highest concentrations in oolong tea and dark tea were further selected for analysis to observe more intuitively the dynamic changes of volatiles during treatment with graphene heating film. In the study of oolong tea, it can be found that almost all the concentrations of the highest volatile substances were basically consistent with the total volatile substances during the graphene heating film treatment, with the peaks at M10 and H10, and a significant decrease in the concentrations of all volatile substances under other treatments (Fig. 3e-m). In dark tea, on the other hand, the concentrations of methyl salicylate, trans-Linalool oxide (furanoid) and trans-β-Ionone were significantly increased relative to CK only at H30, and the other treatment groups did not significantly enhance the concentrations of the nine selected volatile substances, and even some treatments caused the concentrations of these volatile substances, and some treatments even caused a decrease in the concentration of these volatile substances (Fig. 4e-m). The application of the graphene heating film has been demonstrated to enhance the aroma of oolong tea, with the M10 and H10 settings offering optimal results. For dark tea, the H30 setting or longer may be necessary to achieve the desired aroma enhancement.

#### 3.2.2 Analysis of aroma types of oolong tea and dark tea after graphene heating film processing

The volatiles identified in this study were subsequently categorized based on their aroma types. The aroma of these volatile substances can be broadly categorized into floral, fruity, green, baking, and woody types. The total concentrations of volatile metabolites from these five categories of definite aroma types were aggregated to create an aroma flavor radar chart (Fig. 3d and 4d). The analysis revealed that the overall aroma of oolong tea is more prominent in floral and roasted aroma, while dark tea is more prominent in floral and fruity aroma. The radar charts demonstrate that the total concentration of floral and roasted aroma peaked in the M10 and H10 treatments for oolong tea, and the total concentration of floral and fruity aroma peaked in the H30 treatment for dark tea. The findings of this study indicate that the application of graphene heating film at temperatures of M10 or H10 significantly enhances the overall aroma of oolong tea, while the treatment of H30 significantly enhances the overall aroma of dark tea.

#### 3.2.3 Identification and analysis of characteristic aroma in different tea types after graphene heating film processing

**Fig. 5:**
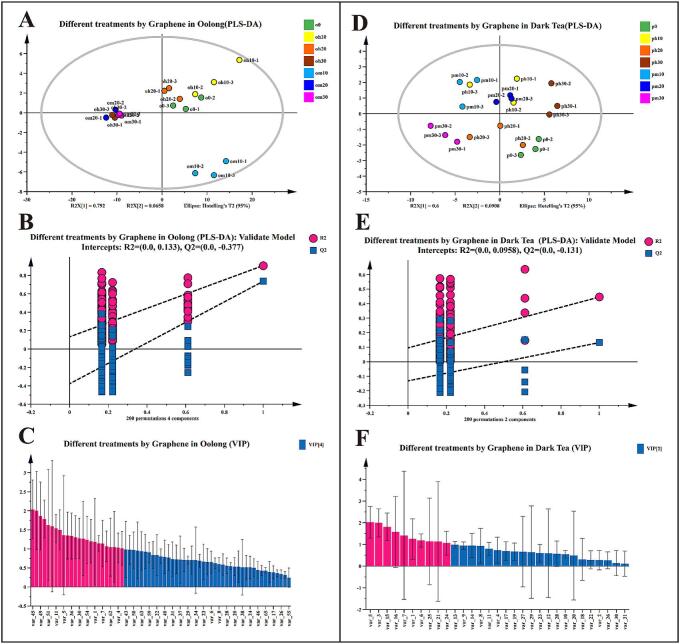
Multivariate statistical analysis of the volatile components of oolong tea and dark tea treated with graphene heating film at different temperature grades and time. (A) PLS-DA score plot of the volatile components of oolong tea; (B) Cross-validation results: the intercept of the Q2 replica line of the cross-validated model for 200 comparisons was less than 0, which indicated that there was no overfitting in the PLS-DA discriminant model, and that the model was relatively reliable; (C) VIP score plot: the pink color indicated the volatile compounds with VIP > 1; and the blue color indicated the volatile compounds with VIP < 1; (D) PLS-DA score plot of volatile components of dark tea; (E) Cross-validation results: the intercept of the Q2 replica line of the cross-validated model for 200 comparisons is less than 0, indicating that the PLS-DA discriminant model is not overfitted and the model is relatively reliable; (F) VIP score plot: pink bars indicate volatile compounds with VIP > 1; blue color indicates volatile compounds with VIP < 1. (For interpretation of the references to color in this figure legend, the reader is referred to the web version of this article.)

In order to further identify and analyze the characteristic volatile components of graphene heating film on oolong tea and black tea processing, PLS-DA models were constructed based on 68 volatile compounds in oolong tea and 31 volatile compounds in dark tea, respectively. The PLS-DA analyses of volatiles after different treatments with graphene heating film were constructed for the two different tea types, and the models constructed for the two tea types were suitably predictable with no overfitting (Fig. 5B, E). In terms of specific tea types, in oolong tea, 24 volatile components with VIP > 1, including 5 alcohols, 2 ketones, 3 esters, 5 aldehydes, and 9 other volatile components (Fig. 5C); in dark tea, 10 volatile components with VIP > 1, including 3 alcohols, 1 ketone, 3 esters, 1 aldehyde, and 2 other volatile components (Fig. 5F).

The contribution of a volatile substance to the overall aroma is related to its concentration and Odor Activity Value (OAV), which refers to the minimum concentration at which a compound has a perceptible odor. Volatile components that satisfy the conditions of VIP > 1 and OAV ≥ 1 and whose aroma has been identified were selected as potential key aroma substances. Based on the results of comparing the calculated RI with the RI of the NIST system, it can be seen that all the potential key aroma substances identified are reliable (Table S1-S2).

Fifteen aroma components were identified in the oolong teas treated with different temperature grades and processing times of graphene heating film, including four floral aroma, three woody aroma, one grassy aroma, and two citrus aroma (Table 1). During processing, seven substances were identified as potential key aroma compounds: trans-Isoeugenol, Nerolidol, Benzyl nitrile, Cedrol, Indole, Limonene, and Benzene (2-nitroethyl). The study demonstrated that oolong tea significantly enhanced the floral and woody aromas after M10 or H10 treatment. However, the enhancement of these related aromas was observed to decrease with processing times of 20 min (M20, H20) and 30 min (M30, H30), reaching levels lower than those observed in tea samples without reheating.

**Table 1:** Fifteen aroma components identified in oolong tea.

Volatile Compounds	Odor Type	VIP	OT (μg/kg)	OAVs						
				CK	M10	M20	M30	H10	H20	H30
2-Furancarboxaldehyde, 5-methyl-	Sweet	2.04	500^1^	0.011	0.033	0.005	0.008	0.018	0.012	0.014
Benzaldehyde	Sweet	2.01	350^1^	0.007	0.006	0.002	0.003	–	0.005	0.008
trans-Isoeugenol	Floral	1.86	0.71^2^	4.855	–	2.855	2.796	7.195	4.327	4.286
Nerolidol	Floral	1.79	0.25^1^	182.598	201.635	77.435	84.460	223.886	152.794	126.164
3-Hexen-1-ol benzoate	green	1.63	500^1^	–	0.008	0.002	0.002	0.005	0.003	0.004
Benzyl nitrile	Aromatic	1.54	1^3^	29.624	33.166	11.663	13.891	32.698	25.571	20.341
2,6-Octadien-1-ol,3,7-dimethyl-	citrus	1.50	49^1^	0.042	0.046	0.007	0.015	–	0.019	0.024
Phenylethyl Alcohol	Floral	1.35	390^1^	0.051	0.050	0.019	0.021	0.056	0.041	0.043
Cedrol	Woody	1.34	0.5^2^	7.209	9.887	2.721	2.293	5.743	2.600	5.182
Caryophyllene	Woody	1.31	64^1^	–	0.039	0.009	–	0.026	0.021	–
Indole	Floral	1.14	0.04^4^	136.634	173.782	86.854	86.624	217.017	172.748	186.263
1H-Pyrrole-2-carboxaldehyde, 1-ethyl-	Roasted	1.14	37^5^	0.390	0.402	0.136	0.163	0.426	0.293	0.232
(3*R*,6*S*)-2,2,6-Trimethyl-6-vinyltetrahydro-2H-pyran-3-ol	woody	1.07	3000^6^	0.001	0.001	0.001	0.001	0.002	0.002	0.001
Limonene	Citrus	1.03	2.5^7^	1.003	1.246	0.273	0.446	0.767	0.412	0.803
Benzene,(2-nitroethyl)-	Aromatic	1.01	2^7^	4.246	4.076	1.796	2.321	4.737	4.008	3.684

Data sources: ^1^(Guo, Ho, Schwab, & Wan, 2021), ^2^(Zhai et al., 2022), ^3^(Hou et al., 2020), ^4^(Zhu et al., 2015), ^5^(Wang et al., 2022), ^6^(Zhang et al., 2021), ^7^(Fang et al., 2023). “--” indicates that the substance was not detected in the sample.

Similarly, six aroma components were identified in the dark tea treated with different temperature levels and processing times of the graphene heating film, including four floral, one fruity, and one grassy aroma (Table 2). After graphene heating film processing, three kinds of potential key aroma substances were qualified, which were Linalool, trans-β-Ionone and Decanal. The results showed that H30 enhanced the floral aroma of dark tea most significantly. And no significant fruity aroma (Decanal) could be identified in the mid-range treatments (M10, M20, M30).

**Table 2:** Six aroma components identified in pu-erh tea.

Volatile Compounds	Odor Type	VIP	OT (μg/kg)	OAVs						
				CK	M10	M20	M30	H10	H20	H30
Terpineol	Floral	2.02	330^1^	0.032	0.012	0.020	0.014	0.020	0.023	0.030
Linalool	Floral	1.99	0.22^2^	59.394	22.973	38.605	24.542	41.610	43.720	60.677
trans-β-Ionone	Floral	1.81	0.01^2^	540.428	443.021	620.386	188.749	576.928	428.734	724.927
Decanal	Fruity	1.42	3^3^	1.251	0.541	0.969	0.366	0.494	1.274	0.585
Methyl salicylate	Green	1.18	40^2^	0.125	0.059	0.114	0.049	0.092	0.094	0.161
trans-Linalool oxide (furanoid)	Floral	1.06	60^4^	0.064	0.037	0.062	0.027	0.049	0.055	0.094

Data sources: ^1^(Guo, Ho, Wan, et al., 2021), ^2^(Li et al., 2024), ^3^(Fang et al., 2023), ^4^(Yang et al., 2023).

#### 3.2.4 Changes of characteristic aroma in oolong tea and dark tea during graphene heating membrane processing

We used the potential key aroma substances identified in the above analytical methods to further analyze the changes of these substances' content under the treatment of graphene heating film at different gears and different times (Fig. 3i-s; Fig. 4k-n). As can be seen from the figure, in oolong tea, the concentrations of nerolidol, benzyl nitrile, cedrol, indole and limonene were enhanced after M10 treatment, among which the concentrations of cedrol and limonene were significantly increased, while in M20 and M30 treatments, the concentrations of all the characteristic aroma substances were significantly decreased. In H10, the concentrations of trans-isoeugenol, nerolidol, benzyl nitrile, indole, and benzene, (2-nitroethyl)- were increased, with a significant increase in the concentrations of trans-isoeugenol and indole; similarly, in H20 and H30 treatments, the concentrations of all the characteristic aroma substances decreased to different degrees in H20 and H30 treatments (Fig. 3i-s). These trends were consistent with the above trends in the OAV values of the characteristic aroma compounds.

The application of graphene heating film treatments at different gears and times did not yield the same results for oolong tea as it did for dark tea. The concentrations of all characteristic aroma substances in dark tea decreased under M10 and M30 treatments, while the concentration of trans-β-Ionone increased to a certain extent under M20 treatment. Among the high-grade treatments, only H30 demonstrated a significant increase in trans-β-Ionone concentration in dark tea. The concentrations of characteristic aroma substances exhibited either no change or only a negligible effect under the other treatments (H10 and H20). Consequently, for dark tea, the H30 treatment exhibited the optimal performance in terms of the comprehensive consideration of the characteristic aroma substances (Fig. 4k-n).

After heating, compared with CK, H10 treatment significantly increased the content of floral substances trans-Isoeugenol and Indole in the potential key aroma components of oolong tea, and the wood aroma substance Cedrol and fruity substance Limonene were significantly enhanced under M10 treatment. H30 treatment significantly enhanced the content of floral substance trans-β -Ionone content.

## 4 Discussion

Heating of tea leaves using graphene heating film showed no significant changes in caffeine and no significant differences in the total content of catechin monomers except for M10 (oolong tea) and H20 (dark tea), but heating promoted the changes in ester-type catechin and non-ester-type catechin contents, which could further balance the ester-type catechins and non-ester-type catechins. The optimal heating parameters were different for different tea types. For oolong tea, the most significant decrease in ester catechin content and increase in non-ester catechin content was at M10 or H10 (Fig. 2A). For dark tea, the most significant decrease in ester catechin content and increase in non-ester catechin content was at H20 and M20 (Fig. 2B). The ester catechins were bitter and astringent with strong astringency, while the non-ester catechins were weak in astringency and mainly contributed to the taste of the tea broth. The decrease of ester catechins and the increase of non-ester catechins after heating could improve the quality of tea to some extent, attenuate the bitterness and astringency of tea broth, and enhance the sweetness.

**Fig. 6:**
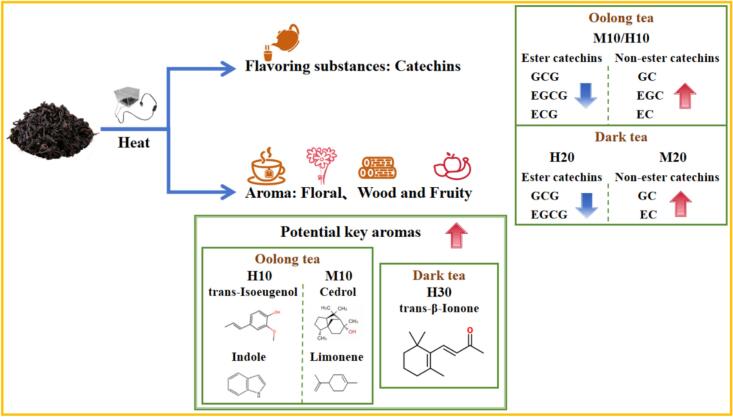
Schematic diagram of sensory evaluation and aroma enhancement of oolong tea and dark tea by graphene heating film.

There were also differences in the effects of graphene heating film on the aroma of different tea types. After heating, the floral, green, woody, roasted and fruity aromas of oolong tea were enhanced, and all of them were most significantly enhanced at the parameter of 10 min of heating (M10, H10) (Fig. 3d). For dark tea, it mainly reduced the green aroma and increased the floral, woody and fruity aroma, and the content of floral, woody and fruity aroma was at the highest level under the parameter of H30 (Fig. 4d). (See Fig. 6.)

Graphene heating film treatment had a large effect on the aroma substances, and there were differences in the effects on different tea types. After the heating treatment with graphene heating film, the types of aroma substances of tea increased, and the relative contents of some major aroma substances were also enhanced more significantly. For oolong tea, the graphene heating film for tea aroma enhancement is more regular, as can be seen from the results of this study, after heating, oolong tea's floral aroma, green aroma, wood aroma, baking aroma and fruity aroma were enhanced, and all of them were in the parameter of the heating for 10 min (M10, H10) oolong tea characteristics of the concentration of the aromatic substances is at the highest level (Fig. 3d), in which the Cedrol, Limonene, trans-Isoeugenol, Indole and other woody and fruity aroma substances were significantly enhanced, which made the tea aroma more intense (Fig. 3i-s). For dark tea, it mainly decreased the green aroma and increased the floral, woody and fruity aromas, and the contents of floral, woody and fruity aromas were at the highest level under the parameter of H30, with the significant enhancement of trans-β-Ionone (Fig. 4l). With the increase of heating intensity, the aroma types of both tea types, oolong tea and dark tea, were transformed to floral, fruity and woody aroma, but the changes of aroma content were different, which indicated that the enhancement effect of graphene heating film on tea quality was related to tea types. In this study, Rougui tea and puerh tea were selected as representatives of oolong tea and dark tea, respectively, to investigate the enhancement effect of graphene heating film treatment on these two types of teas. However, the study did not extend to other types of teas, such as white, green, yellow, and black teas, which were treated with graphene heating film.

Under the same heating time, there was no significant difference in the biochemical compositions of caffeine and catechin in oolong tea and dark tea between medium and high heating gears, while there was a significant difference in the biochemical compositions of tea under different heating times at the same heating gear. The trend of the effect of gear and time on aroma was also basically consistent with the trend of biochemical composition, with insignificant changes in aroma content at different gears at the same time and significant changes in aroma content at different times at the same gears, and the trend of aroma changes was similar with the increase of heating time under medium and high gears. This shows that the heating gear of graphene heating film has less influence on the flavor and aroma of oolong tea and dark tea than the heating time.

In summary, if one aims to enhance the flavor and aroma of tea, it is possible to apply a graphene heating film to oolong tea for a period of 10 min or to dark tea for 30 min using a high-temperature heating method. This approach has been shown to reduce the ester catechin content while increasing the levels of non-ester catechins and potential key aroma substances. In oolong tea, the content of GCG, EGCG and ECG was reduced, the content of GC, EGC and EC was increased, and the floral aroma substances trans-Isoeugenol, Nerolidol, Indole and Benzene, (2-nitroethyl)-; The presence of the baking aroma substance Benzyl nitrile; and the wood aroma substance Cedrol was also identified. In dark tea, the content of GCG and EGCG was reduced, the content of GC and EC was increased, and the floral aroma substances Linalool and trans-β-Ionone were enhanced.

## 5 Conclusion

In this study, oolong tea and dark tea were subjected to graphene heating film at medium (65 °C) and high (75 °C) grades for 10, 20, and 30 min, respectively. The findings revealed a substantial reduction in the ester catechins (GCG, EGCG, ECG) of oolong tea treated with graphene heating film when M10 or H10 was selected. Conversely, the concentrations of non-ester catechins (GC, EGC, EC) and related characteristic aroma substances were found to be significantly increased when M10 or H10 was selected. Specifically, the concentrations of aroma compounds such as Cedrol and Limonene were found to be significantly increased under M10 treatment, thereby enhancing the woody and fruity aroma of tea. In contrast, H10 treatment led to a significant increase in the concentrations of aroma compounds like trans-Isoeugenol and Indole, contributing to the enhancement of the floral aroma of tea. Conversely, the content of ester catechins (GCG, EGCG) of dark tea decreased at H20, while the content of non-ester catechins (GC, EC) increased at M20. Additionally, the concentration of the characteristic aroma substance trans-β-Ionone was enhanced at H30, contributing to the floral aroma of tea. In summary, the application of graphene heating film to enhance tea aroma should be tailored to the specific tea type of the tea samples to select the appropriate temperature level and processing time to obtain the best enhancement effect. This study also confirms that graphene heating film can be used as a tool to enhance tea aroma, providing a theoretical basis for the application of new technology and equipment for tea aroma enhancement.

## CRediT authorship contribution statement

**Jiyuan Yao:** Writing – original draft, Validation, Methodology, Investigation, Formal analysis, Data curation, Conceptualization. **Xinyuan Lin:** Writing – original draft, Formal analysis, Data curation, Conceptualization. **Zihao Qiu:** Methodology, Investigation. **Xun Meng:** Validation. **Juan Chen:** Validation. **Ansheng Li:** Investigation. **Xindong Tan:** Writing – review & editing, Supervision, Resources, Project administration, Methodology. **Shaoqun Liu:** Writing – review & editing, Resources, Project administration. **Peng Zheng:** Writing – review & editing, Resources, Project administration. **Binmei Sun:** Resources, Project administration. **Hongqiang Kong:** Resources, Project administration, Methodology.

## Declaration of competing interest

The authors declare that they have no known competing financial interests or personal relationships that could have appeared to influence the work reported in this paper.

## Data Availability

Data will be made available on request.

## Appendix A Supplementary data

Supplementary material Image 1
