# Supplementary material for: Enhancement of flavor components of oolong tea and dark tea based on graphene heating film
Source: Food Chem X. 2025 Apr 3;27:102433. doi: 10.1016/j.fochx.2025.102433 (PMC12002625; doi:10.1016/j.fochx.2025.102433)
Supplement: Supplementary file 1 — Supplementary material [file mmc1.docx]

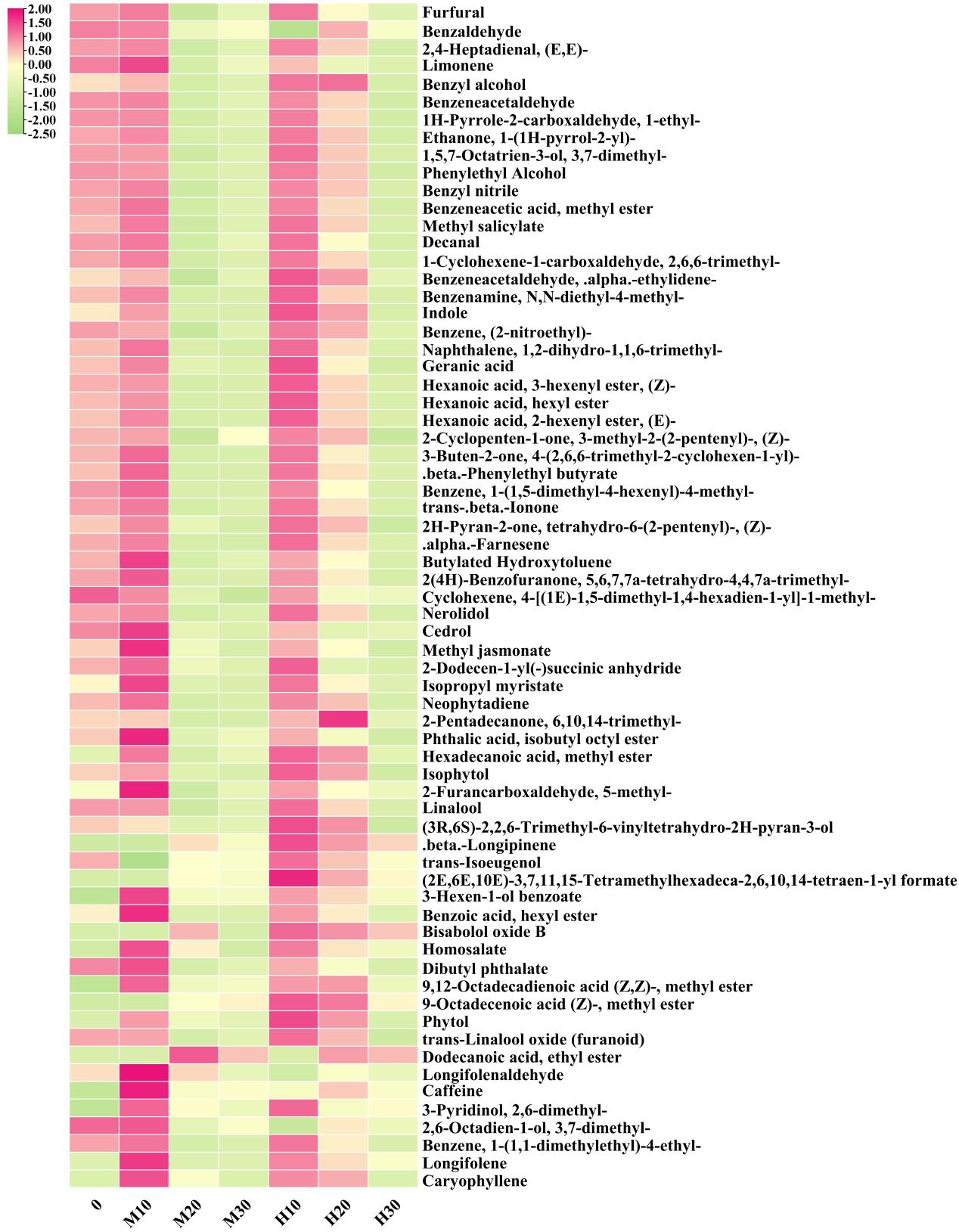


**Figure S1** Heat map of the changes in the concentration of volatile compounds of oolong tea under different treatments, n=3.


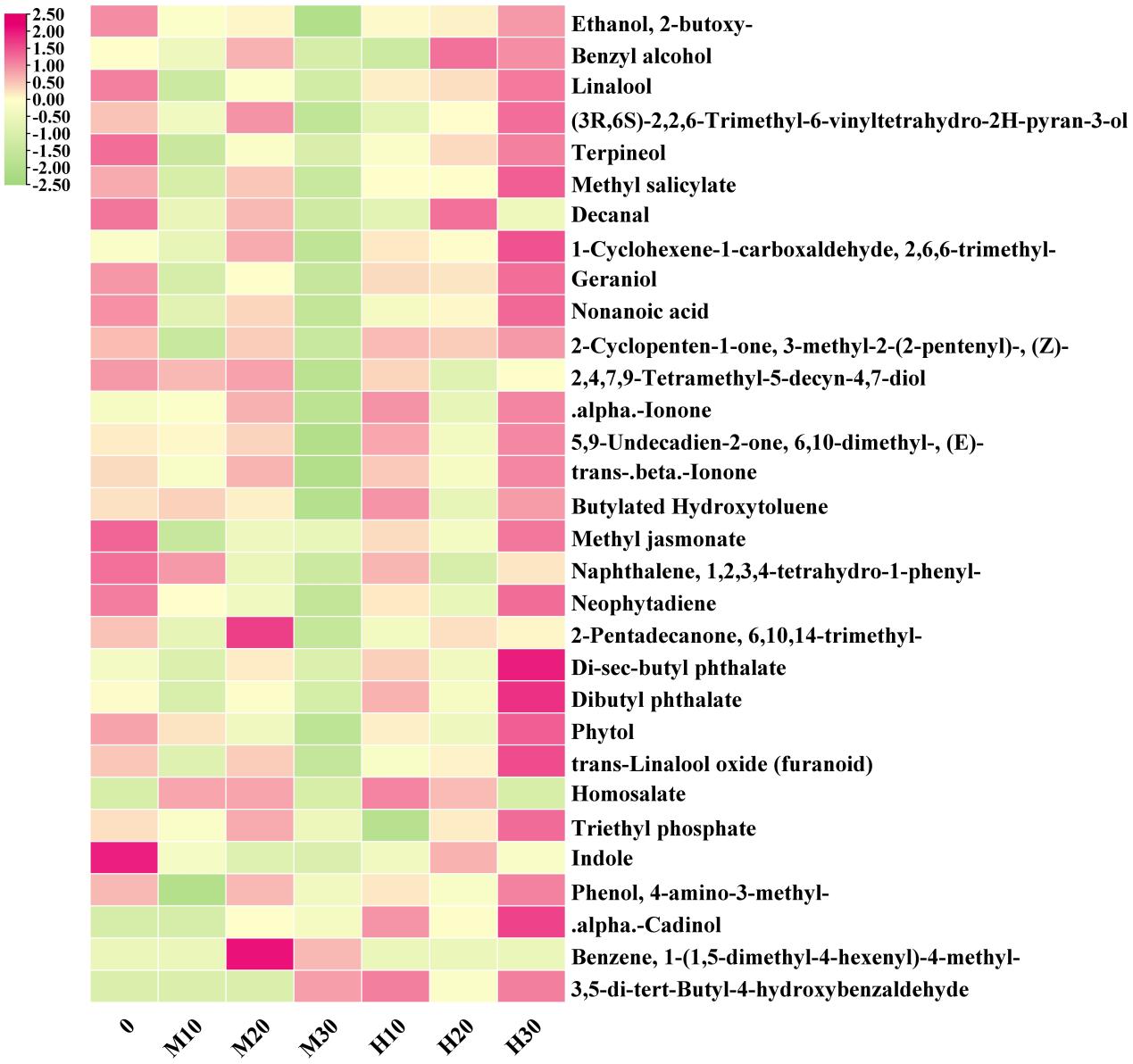


**Figure S2** Heat map of the changes in the concentration of volatile compounds of dark tea under different treatments, n=3.

**Table S1** Qualitative results of fifteen aroma components in oolong tea

| **Volatile Compounds** | **RI^a^/RI^b^** | **Volatile Compounds** | **RI^a^/RI^b^** | **Volatile Compounds** | **RI^a^/RI^b^** |
| --- | --- | --- | --- | --- | --- |
| 2-Furancarboxaldehyde, 5-methyl- | 992/966 | Benzyl nitrile | 1137/1143 | Indole | 1291/1293 |
| Benzaldehyde | 965/961 | 2,6-Octadien-1-ol, 3,7-dimethyl- | 1251/1245 | 1H-Pyrrole-2-carboxaldehyde, 1-ethyl- | 1046/1046 |
| trans-Isoeugenol | 1447/1449 | Phenylethyl Alcohol | 1111/1121 | (3R,6S)-2,2,6-Trimethyl-6-vinyltetrahydro-2H-pyran-3-ol | 1166/1183 |
| Nerolidol | 1561/1535 | Cedrol | 1600/1608 | Limonene | 1024/1020 |
| 3-Hexen-1-ol benzoate | 1568/1568 | Caryophyllene | 1463/1451 | Benzene, (2-nitroethyl)- | 1296/1305 |

**“ RI^a^ ”** means RI calculated on the basis of substance retention time, **“ RI^b^ ”** means the RI of the corresponding substance queried in the NIST system.

**Table S2** Qualitative results of six aroma components in dark tea

| **Volatile Compounds** | **RI^a^/RI^b^** | **Volatile Compounds** | **RI^a^/RI^b^** | **Volatile Compounds** | **RI^a^/RI^b^** |
| --- | --- | --- | --- | --- | --- |
| Terpineol | 1187/1190 | trans-.beta.-Ionone | 1482/1498 | Methyl salicylate | 1192/1187 |
| Linalool | 1096/1104 | Decanal | 1201/1200 | trans-Linalool oxide  (furanoid) | 1069/1069 |

**“ RI^a^ ”** means RI calculated on the basis of substance retention time, **“ RI^b^ ”** means the RI of the corresponding substance queried in the NIST system.
